# Modeling Alpha-Band Functional Connectivity for MEG Resting State Data: Oscillations and Delays in a Spiking Neuron Model

**DOI:** 10.1186/1471-2202-14-S1-P99

**Published:** 2013-07-08

**Authors:** Tristan T Nakagawa, Henry Luckhoo, Mark Woolrich, Morten Joensson, Hamid Mohseni, Morten Kringelbach, Viktor Jirsa, Gustavo Deco

**Affiliations:** 1 Department of Technology, Universitat Pompeu Fabra, Barcelona, Spain; 2 Centre for Human Brain Activity, University of Oxford, Oxford, UK; 3 Department of Psychiatry, University of Oxford, Oxford, UK; 4 FCIN, Aarhus University, Denmark; 5 CNRS, Université de la Méditeranée, Marseille, France; 6 ICREA, Barcelona, Spain

## 

The study of structural and functional connectivity (SC,FC) and dynamics in spontaneous brain activity is a rapidly growing field of research [1]. The existence of Resting State Networks (RSN) has been well established in fMRI over the past decade, [1] and computational models [2] have successfully captured their connectivity patterns and slow oscillations, but have not been applied to recent MEG findings of coherent RSN [3] yet.

Here, we extended a recent neurophysiologically realistic spiking-neuron model of spontaneous fMRI activity [4] to exhibit noisy oscillatory activity in the alpha band (Figure 1A, bottom) and studied how connectivity and delays influenced the model fit with the oscillatory MEG FC. The global network was described by a graph of nodes (local populations of excitatory and inhibitory spiking neurons), connected to each other according to a DTI-derived anatomical connectivity matrix, which fixed the relative connectivity and delay/distance structure, but left global scaling factors W (coupling weight) and ps (propagation speed in m/s) as free parameters in the model. FC was measured by correlating the low-pass filtered Power Envelopes of the bandlimited signal. Simulations showed the largest margin of good concordance with empirical FC over W when neurophysiologically realistic delays (5-10 m/s) were included (Figure 1C).

**Figure 1:**
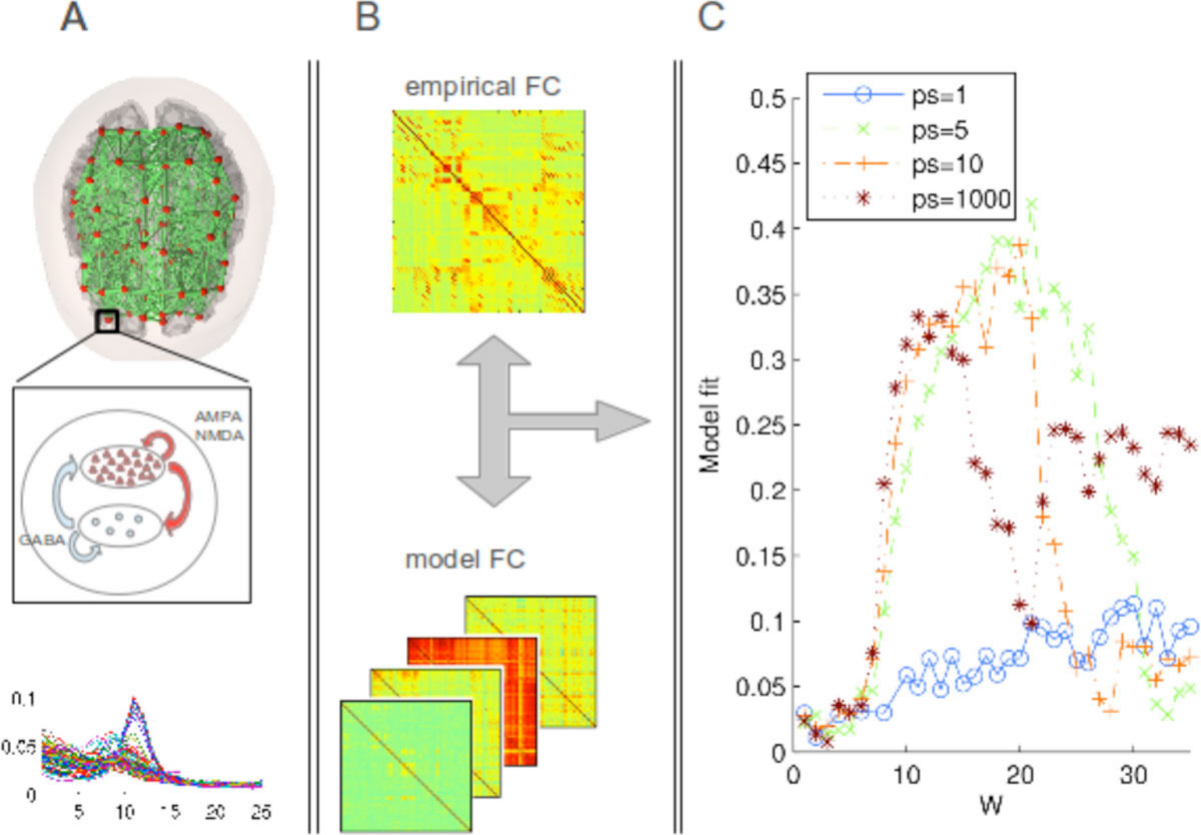
**A: Sketch of the global model graph, each node consisting of local populations of spiking neurons**. The model is capable of producing alpha oscillations (bottom). B: Empirical and simulated FC are fitted and C: the model best captures the empirical pattern with physiological delays (ps = 5-10 m/s).

## Conclusions

In the presence of noisy oscillations on the same order of magnitude as system delays, the temporal connectivity structure plays a role in shaping the functional network connectivity. By effectively decreasing strong synchronous inputs to nodes, the network is stabilized and the need for fine-tuning of global coupling reduced when compared to the absence of delays.
